# Potential Impact of Dietary Choices on Phosphorus Recycling and Global Phosphorus Footprints: The Case of the Average Australian City

**DOI:** 10.3389/fnut.2016.00035

**Published:** 2016-08-26

**Authors:** Geneviève S. Metson, Dana Cordell, Brad Ridoutt

**Affiliations:** ^1^Institute for Sustainable Futures, University of Technology Sydney, Ultimo, NSW, Australia; ^2^Commonwealth Scientific and Industrial Research Organisation, Clayton South, VIC, Australia; ^3^Department of Agricultural Economics, University of the Free State, Bloemfontein, South Africa

**Keywords:** phosphorus, diet, footprint, sustainable resource use, recycling, Australia

## Abstract

Changes in human diets, population increases, farming practices, and globalized food chains have led to dramatic increases in the demand for phosphorus fertilizers. Long-term food security and water quality are, however, threatened by such increased phosphorus consumption, because the world’s main source, phosphate rock, is an increasingly scarce resource. At the same time, losses of phosphorus from farms and cities have caused widespread water pollution. As one of the major factors contributing to increased phosphorus demand, dietary choices can play a key role in changing our resource consumption pathway. Importantly, the effects of dietary choices on phosphorus management are twofold: First, dietary choices affect a person or region’s “phosphorus footprint” – the magnitude of mined phosphate required to meet food demand. Second, dietary choices affect the magnitude of phosphorus content in human excreta and hence the recycling- and pollution-potential of phosphorus in sanitation systems. When considering options and impacts of interventions at the city scale (e.g., potential for recycling), dietary changes may be undervalued as a solution toward phosphorus sustainability. For example, in an average Australian city, a vegetable-based diet could marginally increase phosphorus in human excreta (an 8% increase). However, such a shift could simultaneously dramatically decrease the mined phosphate required to meet the city resident’s annual food demand by 72%. Taking a multi-scalar perspective is therefore key to fully exploring dietary choices as one of the tools for sustainable phosphorus management.

## Introduction: The Global Phosphorus Challenge

Food production, and as such human diet, is the main driver of global phosphorus demand. Phosphorus is almost exclusively mined for food production (1), but globally, only 20% of what is mined as phosphorus is ultimately consumed as food (2). Phosphorus is essential for all life and has no substitute in plant and animal growth. However, different foods types require different amounts of phosphorus, which are sourced naturally from soils or in the form of fertilizers, feed, and food additives. Part of these different requirements stem from different crop and animal nutrient needs. These different requirements also stem from the additional losses^1^ of phosphorus that occur at each step of the food chain. Phosphorus can be lost or wasted through runoff and erosion from fields, as part of crop and food waste, as well as through the excreta of animals and humans. The process of having to grow animal feed crops, rearing animals, and converting them to human food requires substantially more land, energy, and water than the direct consumption of crops by humans (3), and this is also the case for phosphorus (4). Globally, 28% of the increased phosphate fertilizer consumption over 46 years (1961–2007) can be attributed to changes in diet, making it not only an important driver of global phosphorus flows but also a potential leverage point to increase phosphorus sustainability (4). As global population and per capita meat consumption increase, so will the demand for food and hence phosphorus, which could be unsustainable under a “business as usual” scenario (5).

Phosphorus management is emerging as a serious global challenge to ensure affordable and accessible food and clean water. Mined phosphate rock, the source of most phosphorus fertilizers, is a non-renewable global resource, which is becoming an increasingly geopolitically, economically, and physically scarce resource (6). This makes short- and long-term availability and affordability for farmers (and thus consumers) a growing concern (7). We have already experienced some of the effects of short-term phosphorus scarcity, including phosphate fertilizer price spikes, during the 2008 food crisis where the most affected populations were, and continue to be, the ones who need phosphorus the most, such as farmers in developing countries who lack access to sufficient phosphorus to meet crop needs, especially in areas where highly weathered soils with high phosphorus fixation capacity make it more difficult for plants to access the little phosphorus that is applied (8–10).

At the same time, losses of phosphorus to waterways, whether from agricultural fields or urban sewage, can cause severe water quality degradation, resulting in eutrophication, harmful algal blooms, and hypoxia that impair drinking water, recreational areas, and fisheries (11–13). Over 400 coastal water bodies globally are hypoxic because of excess phosphorus and nitrogen additions (14), while 60% of medium and large lakes and reservoirs are eutrophic (15). Current global phosphorus management, or lack thereof, is thus not sustainable (16, 17). The second UN Sustainable Development Goal^2^ highlights that long-term global food security and adequate human nutrition are dependent on agricultural and food chain practices and the resources those practices depend on. Although the goal itself does not mention phosphorus explicitly, as an essential agricultural input phosphorus should be considered in the way we meet this goal. Explicitly considering food system interventions that address phosphorus security will be essential to meeting food security and healthy environment objectives (18).

In this paper, we explore one potentially important intervention to reduce the demand for mined phosphorus, reduce losses to waterways, and influence the recycling potential of waste: human diet. Here, we define diet as the quantity and diversity of food consumed per capita in 1 year, more specifically, the amount of food consumed in 16 basic food categories.^3^ We explore the implications of diet on phosphorus sustainability using the Australian context as a case study. Specifically, we investigated the impact of city residents shifting toward plant-based diets (see Box 1) on mineral fertilizer demand as well as on the potential supply of phosphorus from recycled wastes in urban areas.^4^ We choose the city as the point of intervention because most of the world’s population now live in cities, and in Australia, 90% already live in urban areas. Through this Australian case study, we demonstrate that a multi-scalar viewpoint, from local resource recovery to global mined phosphorus requirements, may change how one views human diet as a lever of change in a city.

Box 1 **Calculating phosphorus consumption and the phosphorus footprint associated with the Australian diet.**

To calculate phosphorus consumption in the average Australian diet, we multiplied national average per capita Australian food intake data by the average phosphorus concentrations of food groups (Eq. 1). More specifically, food intake data from the 2011 National Nutrition Survey were converted into 16 basic food equivalent categories using the same methods as with 1995 survey data described in Ridoutt et al. (19). The consumption of these 16 food groups, reported in weight per capita (e.g., kilogram of raw fruit equivalent per capita) were then multiplied by the average phosphorus content of each food group as documented in the Food Standards Australia New Zealand database (FSANZ, 20).

(1) $$P_{\text{consumed}}=\sum {\;}_{\text{food groups}}{\text{Mass}}_{\text{food group}}\times \;P_{\text{concentration}}$$

(2) $$P_{\text{footprint}}=\sum {\;}_{\text{food groups}}{\text{Mass}}_{\text{food group}}\times \;P_{\text{fertilizer used}}$$

In order to calculate conversion factors for phosphorus content in kilogram of phosphorus per kilogram of ingested food for the vegetable oil, tea and coffee, and alcoholic beverage food groups, an additional step was required. The concentration of phosphorus for these food groups was reported by phosphorus content in one liquid portion size by FSANZ, and thus, we used the density of water to calculate a conversion factor based on food intake weight. For the dairy food group, we used the density of milk (1.03 g/ml) to convert the phosphorus ingested per portion to an average phosphorus content conversion factor. We assume that 98% of phosphorus ingested is excreted (21).

For the plant-based diet, we converted meat, dairy, eggs, and seafood food groups to pulses (i.e. beans or legumes) based on protein equivalent in order to keep the protein intake of the diet identical to 2011 reported levels (20). This may be a conservative estimate, as the average global citizen consumes a third more protein than actually required (22), much greater for Australia, hence a shift to a plant-based diet in Australia would not require replacement of all protein currently consumed. However, the nutritional value of plant-based protein vs. meat-based protein is still debated (23), and some sources recommend increasing protein intake to 125% of the omnivore daily recommendated values, if eating a plant-based diet (24).

The phosphorus footprint calculations were based on the conversion factors developed in Metson et al. (4) (Eq. 2). Instead of the FAO food supply per capita used by Metson et al. (4), we used the 2011 Australian specific food intake plus food waste estimates [using the same methods as described in Ridoutt et al. (19)] to be consistent with the phosphorus consumption numbers described above. The food waste estimates were reported as a percentage for each food group intake weight and then added to intake. The current diet Australian footprint calculated here is lower than the value calculated in Metson et al. (4) (6.51 kg per capita). The discrepancy is likely due to the use of different data, even though, theoretically, both the bottom up (food intake to food grown used here) and the top down (food grown to dietary intake as used by FAO) should be roughly comparable.

## Footprint Analyses, Phosphorus, and Human Diet

Environmental and ecological footprints are used to determine the resources needed to support particular consumption patterns, whether it is for an individual, an economic sector, or a community (25, 26). The intent is to better assess the impact of particular practices on sustainability, both from a resource use and pollution perspective. Dietary choices and agricultural practices, for example, have been studied for their water (27), energy and land (3), climate change contribution (28, 29), nitrogen (30), and phosphorus (4) footprints, both locally and globally. Footprint analyses date back to the 1990s (25), but phosphorus has been underrepresented in footprint analyses until recently [as presented in Metson et al. (31), for example].

Over the past 10 years, there have been many important contributions to measuring direct phosphorus flows through the food system (and non-food system) at the global (2), regional (32, 33), national (34–36), city (37, 38), and household (39) scales. More extensive meta reviews of phosphorus substance flow analyses at multiple scales can be found in Ref. (40, 41). Our understanding of the dietary phosphorus footprint has increased as concerns around local and global sustainable phosphorus management have grown along with an increased synthesis of disparate data.

**Figure 1:**
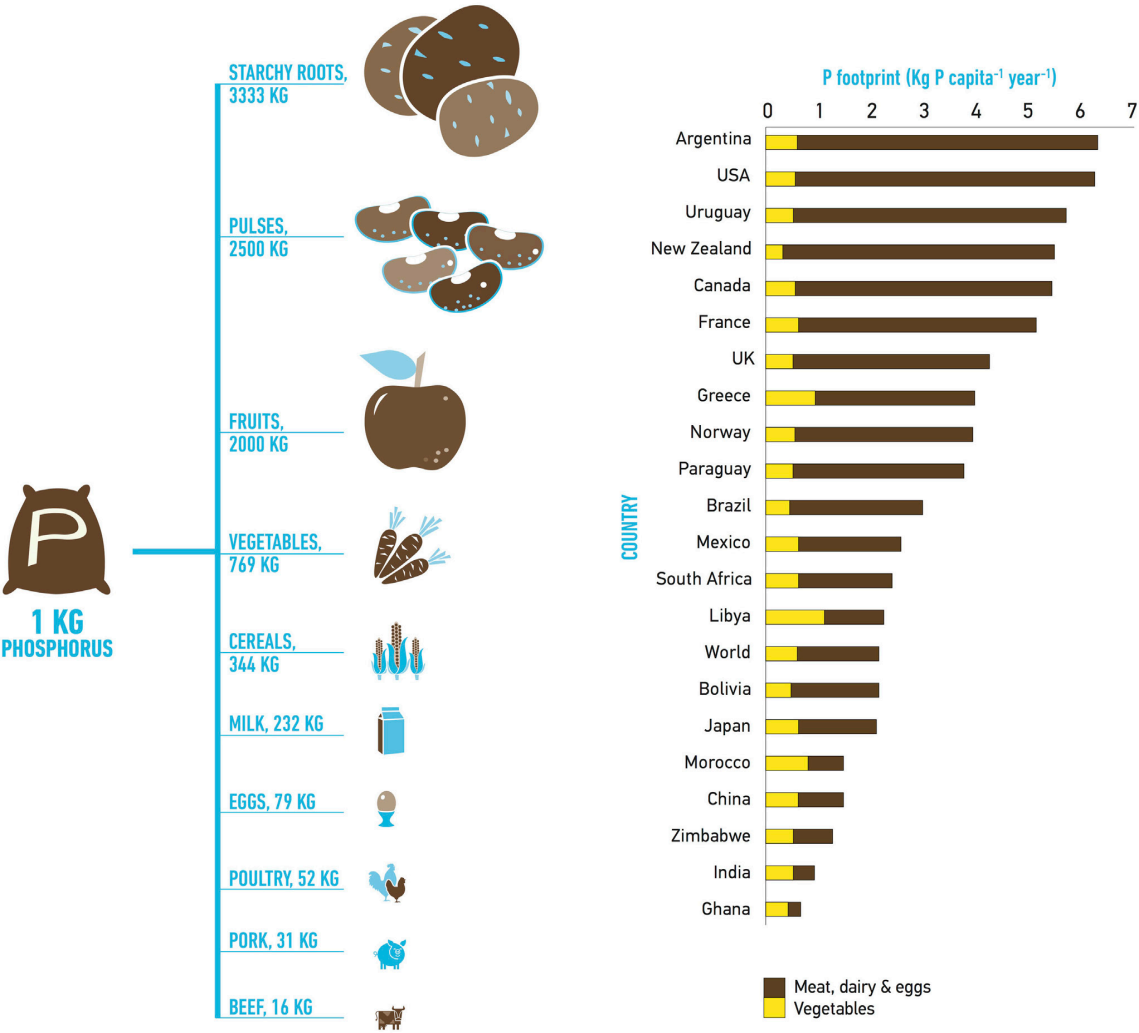
**Dietary phosphorus footprint associated with different food groups and selected countries demonstrating the important contribution of meat to the phosphorus footprint value and the large variability of the phosphorus footprint between countries**. Australia, although not depicted here, has a phosphorus footprint of 6.51 kg per capita. Reproduced with permission from McGill University (42), with data based on Metson et al. (4).

The phosphorus footprint of a country varies, largely due to the amount of meat consumption, as indicated by Figure 1 (4). We define the phosphorus footprint, as in Metson et al. (4), as the total amount of mined phosphorus required to produce the food consumed by one person in a particular country over 1 year. The average per capita human dietary phosphorus footprint has increased 38% globally since 1970 (4). There are important differences in the phosphorus footprint of countries, where countries with high Human Development Index (HDI) values typically consume larger amounts of animal products and have significantly higher phosphorus footprints than lower HDI counties. Although still not as high as the phosphorus footprints of the USA or Argentina, emerging economies have shown rapid phosphorus footprint increases over time (e.g., China’s phosphorus footprint has increased by 400% since 1970).

Some countries with poor nutritional status associated with undernourishment need to increase their caloric intake and diversify their diet, consequently increasing their phosphorus footprint. However, decreasing (or limiting) meat intake in already high phosphorus footprint countries would be an effective strategy to decrease mined phosphorus demand. For example, in the USA, moving from the current meat-intensive diet to a plant-based diet could potentially decrease the country’s phosphorus fertilizer demand by 44% (43). Similarly, a recent study of the Austrian food system revealed that moving toward healthier and less meat-intensive diet would decrease phosphorus demand by 20–25% and decrease phosphorus losses to waterways by 5–6% (44). It is clear that dietary choices have a large impact on the whole food production chain and thus on phosphorus sustainability globally. However, the effect of diet on phosphorus consumption and pollution depends on the particular context of analysis, such as type of livestock systems, source of phosphorus input into livestock systems (fertilized pastures, grain-fed, fodder, additives). For example, in Australia, 63% of the country’s phosphorus demand is associated with livestock production because the majority of animals are reared in fertilized pasture systems rather than intensive feedlots (45).

## The Impact of Diet on Australians’ Phosphorus Footprint and Waste

**Figure 2:**
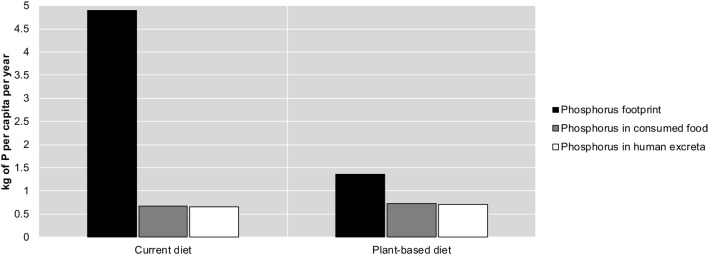
**Comparison of the phosphorus footprint (black), phosphorus in consumed food (gray), and phosphorus in human excreta (white) in the current Australian diet and a hypothetical plant-based diet**.

In Australia, the amount of phosphorus mined to support the current average person’s diet (the phosphorus footprint) is over three times higher than that needed to support a plant-based diet. However, the amount of phosphorus consumed in the average Australian diet is slightly less than in a plant-based diet (Figure 2).^5^ More specifically, Australians currently ingest approximately 0.67 kg per capita each year in food but have a phosphorus footprint of 4.9 kg per capita, over seven times the amount ingested in food, according to our calculations. This Australian footprint value is similar to that of EU countries in 2007 (4).

A shift to a plant-based diet with the same protein content as the current average diet would potentially increase phosphorus ingested (and thus excreted) by 8% (to 0.73 kg per capita) but would decrease the footprint of each Australian to 1.35 kg per capita – a 72% decrease. Mihelcic et al. (46) reported that globally, the phosphorus excreted through urine and feces vary between 0.18 and 0.73 kg per capita per year where diets higher in vegetable proteins result in higher phosphorus concentrations excreted (following the same trend found in our calculations). Importantly, the difference in phosphorus footprint between a plant-based and a conventional (current) diet is greater than the difference between the phosphorus excreted with these different diets. That is, the impact potential of changing diets is significant for reducing mined phosphorus (footprint) and relatively insignificant for changing the phosphorus content of excreta.

## Potential Implications of Dietary Changes as a Strategic Intervention for Phosphorus Security

There are a wide variety of interventions to move toward phosphorus security – changing diets is just one of these. These solutions range from recycling phosphorus in organic wastes, increasing efficiencies along the food chain to reduce phosphorus demand in all sectors, including mining and fertilizer production, agriculture, food production, and consumption (47, 48). These sustainability interventions can extend the longevity of finite phosphate resources, reduce phosphorus pollution, increase efficiency of the whole supply chain, and reduce communities’ and countries’ dependence on imported phosphate from geopolitically risky regions, buffering against price spikes and supply disruptions (8). Some policies, agricultural practices, and waste recovery strategies to increase phosphorus efficiency and recycling have been implemented locally [see some examples in Cordell et al. (49)]. However, selecting the appropriate suite of measures for each unique social–institutional and biophysical context is crucial (50, 51), and diet is no exception. Taking a multi-scalar and systems approach to examining diet as an intervention point is essential, especially for a city.

The sustainability implications of Australian city residents shifting their current diets to plant-based diets are wide-ranging, in terms of phosphorus security benefits and geographical scales, as indicated in Table 1.

**Table 1:** **The multi-scale impact of changing diets in an average Australian city on phosphorus demand, pollution, and recycling**.

		Scale of impact		
		Local	Regional	Global
**Impact of changing diets at the city scale on:**	Phosphorus demand	–	–	Can drastically reduce demand for mined phosphate (*72% reduction*), thereby extending the longevity of the world’s finite phosphate rock resources, and reducing Australia’s dependence on imported phosphate rock
	Phosphorus pollution	No local pollution reduction potential in cities because negligible reduction in phosphorus content of city resident’s excreta/wastewater (*8% increase*)	May be significant because less phosphorus is used in agriculture and livestock (due to the reduced phosphorus footprint), which implies less phosphorus flowing from agricultural soils into waterways in total (assuming other practices remain the same)	*Same as regional impact*
	Phosphorus recycling	Negligible/minimal changes in recycling potential in cities because no reduction in phosphorus content of city resident’s excreta/wastewater (*8% increase*)	–	–

This Australian phosphorus footprint investigation highlights the multi-scalar implications of dietary choice. That is, the need to consider the role of urban consumer behavior beyond city boundaries in order to better account for the full impacts of local decisions, in this case particularly urban ones, on global and local long-term phosphorus security.

In reality, the impacts of dietary choices on phosphorus demand and pollution are complex and require consideration of additional factors beyond categories of consumed food (e.g., meat, vegetables, and grains); however, there are little data available on such impacts. Consumer choices about food can affect farming practices, which in turn affect fertilizer demand as well as phosphorus losses. For example, deciding to adhere to an organic diet would influence the source and amount of fertilizers added to fields and losses to waterways (52, 53), while also potentially resulting in a healthier diet (54).

In addition, the effect of dietary choices may also extend beyond encouraging best management practices on fields and pastures [see Sharpley and Wang (55), for examples of such practices] to include post-harvest losses of food and thus phosphorus. For example, eating a more local diet may minimize losses from storage and transport food spoilage. Local diets may also potentially make it easier to encourage and evaluate sustainable phosphorus farming practices [as shown through analyses of nitrogen in the food system locally (56) and difficulties of doing so globally (57)]. In other words, the *qualitative* decisions about diet, in addition to the *quantitative* decisions about food group consumption, may be important for phosphorus security. As such, we require more detailed information on farming practices, food supply chains, and dietary habits in order to make more locally accurate estimates of the role of dietary changes on mined phosphorus demand as well as phosphorus in waste streams that could potentially be recycled or affect waterways. Broader environmental and health assessment, which incorporates more than just phosphorus, is also necessary to support robust recommendations about the benefits of changes in production systems and diets.

In summary, both phosphorus recycling and phosphorus demand management should be considered in evaluating and combining solutions to the phosphorus challenge; however, this may not always be obvious when studying phosphorus from an urban perspective. Shifting toward a more plant-based diet may not significantly impact urban phosphorus recycling potential but can have a large impact on the demand for mined phosphorus. As shown here using Australia as a case study, a footprint perspective can allow cities to evaluate potential interventions from a different perspective, allowing stakeholders to make more informed decisions about prioritizing solutions that holistically increase phosphorus security.

## Author Contributions

GM and DC designed research and perspective, GM conducted analysis, BR provided diet composition data, GM lead writing effort with contributions from DC and BR.

## Conflict of Interest Statement

The authors declare that the research was conducted in the absence of any commercial or financial relationships that could be construed as a potential conflict of interest.

## Footnotes

^1^Here, by losses, we are referring to phosphorus that is applied to agricultural land that does not end up incorporated in human and animal tissues within a year. Some of these losses are permanent on human time scales, while others are potentially recoverable (especially phosphorus that may be stored in agricultural soils).

^2^https://sustainabledevelopment.un.org/?page=view&nr=164&type=230&menu=2059

^3^Vegetables, fruits, grains, meat, seafood, nuts and seeds, eggs, beans/peas/pulses, dairy, vegetable oil, tea and coffee, alcoholic beverages, cocoa, honey, and sugar.

^4^A related project specifically looks at *Mapping Sydney’s Phosphorus Supply & Demand*, see http://www.p-futurescities.net/sydney-australia/#MappingSydney

^5^See Box 1 for methods and data used to calculate the amount of phosphorus in food and excreta and the phosphorus footprint of Australians.
